# Supplementary material for: Tight Spaces, Tighter Signals: Spatial Constraints as Drivers of Peripheral Myelination
Source: Cells. 2025 Jun 18;14(12):926. doi: 10.3390/cells14120926 (PMC12190299; doi:10.3390/cells14120926)
Supplement: Supplementary file 1 [file cells-14-00926-s001.zip › cells-3647678-supplementary.pdf]

cells

MDPI

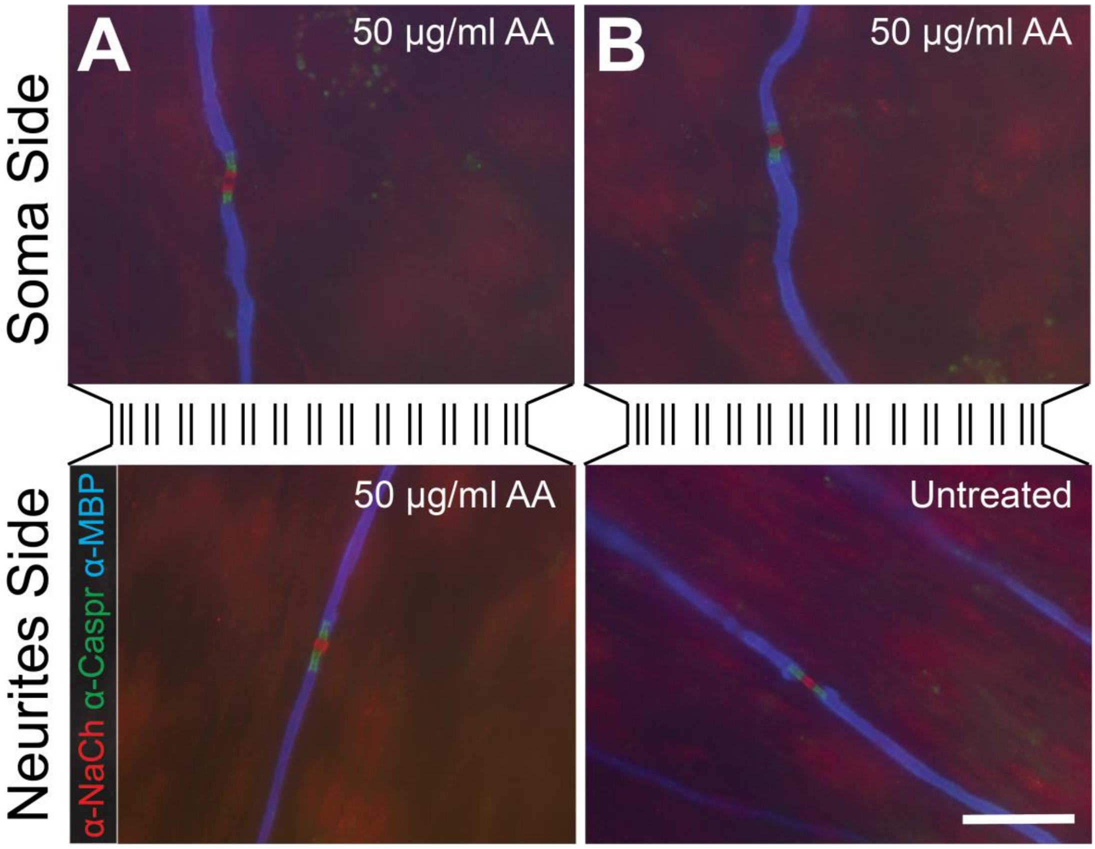

Supplementary Figure S1 - Node of Ranvier Formation.

Immunohistochemical analysis demonstrates that nodes of Ranvier are appropriately assembled in both compartments, irrespective of whether 50 µg/ml AA is applied to both (A) or to only one compartment (B).

Green=Caspr, red=NaCh and blue: MBP. Scale bar: 20 µm.

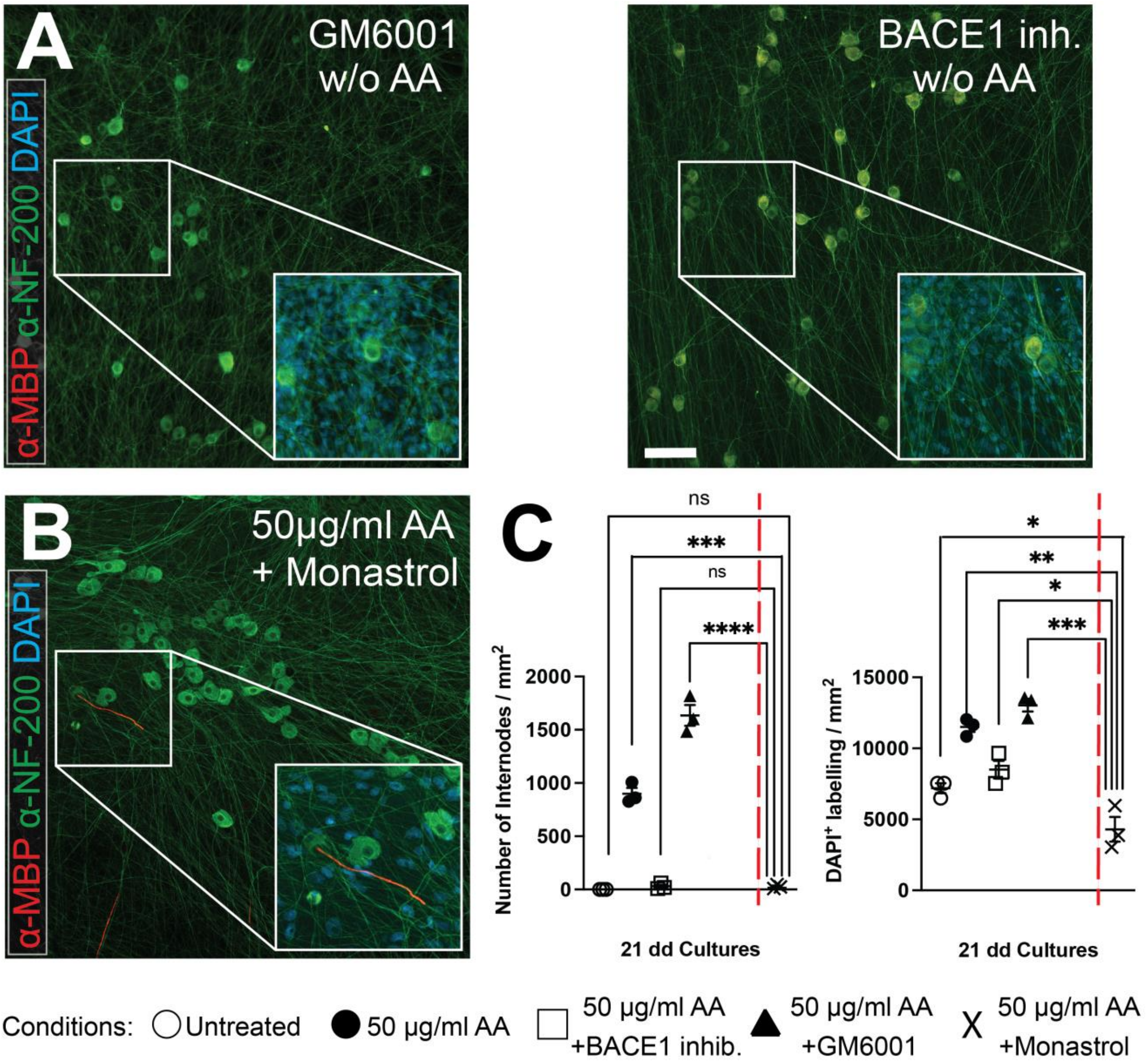

Supplementary Figure S2 - DRG neuron-SC co-cultures treated with secretases inhibitors and antimitotic compounds.

(A) - Immunohistochemical analysis of DRG neuron-SC co-cultures shows that ADAMs inhibitor GM6001 or BACE1 inhibitor without AA do not promote myelination.

(B) - Immunohistochemical analysis of DRG neuron-SC co-cultures co-treated with 50 µg/ml AA and 50 µM Monastrol shows a significant decrease in myelination rate.

(C) - Quantification of internode number and cell count (nuclei) in DRG neurons-SC co-cultures treated with 50 µg/ml AA and 50 µM Monastrol, compared to the conditions shown previously in Figure 2C (left of the red dashed line)**.**

Green=neurofilament, red=MBP; blue: DAPI. Scale bar: 100 µm. For quantifications: n = 3; *p < 0.05, **p < 0.01, ***p < 0.001, ****p < 0.0001, ns: not significant; Student's t test. Data are presented as scatter dot plot with mean ± SEM.
